# Effects of Hydrogen Sulfide Donor NaHS on Porcine Vascular Wall-Mesenchymal Stem Cells

**DOI:** 10.3390/ijms21155267

**Published:** 2020-07-24

**Authors:** Chiara Bernardini, Debora La Mantia, Salvatore Nesci, Roberta Salaroli, Cristina Algieri, Alessandra Pagliarani, Augusta Zannoni, Monica Forni

**Affiliations:** 1 Department of Veterinary Medical Science, University of Bologna, Via Tolara di Sopra, 50-40064 Ozzano Emilia (BO), Italy; chiara.bernardini5@unibo.it (C.B.); debora.lamantia2@unibo.it (D.L.M.); salvatore.nesci@unibo.it (S.N.); roberta.salaroli@unibo.it (R.S.); cristina.algieri2@unibo.it (C.A.); alessandra.pagliarani@unibo.it (A.P.); monica.forni@unibo.it (M.F.); 2 Health Sciences and Technologies—Interdepartmental Center for Industrial Research (CIRI-SDV), Alma Mater Studiorum—University of Bologna, 40100 Bologna, Italy

**Keywords:** H_2_S, gas transmitter, H_2_S donors, vascular wall–mesenchymal stem cells, pig model

## Abstract

Hydrogen sulfide (H_2_S) is now considered not only for its toxicity, but also as an endogenously produced gas transmitter with multiple physiological roles, also in maintaining and regulating stem cell physiology. In the present work, we evaluated the effect of a common H_2_S donor, NaHS, on porcine vascular wall–mesenchymal stem cells (pVW–MSCs). pVW–MSCs were treated for 24 h with increasing doses of NaHS, and the cell viability, cell cycle, and reactive oxygen species (ROS) production were evaluated. Moreover, the long-term effects of NaHS administration on the noteworthy characteristics of pVW–MSCs were analyzed. The MTT test revealed no alteration in cell viability, however, the cell cycle analysis demonstrated that the highest NaHS dose tested (300 μM) determined a block in S phase, which did not depend on the ROS production. Moreover, NaHS (10 μM), continuously administered in culture for 21 days, was able to significantly reduce NG2, Nestin and PDGFR-β expression. The pro-angiogenic attitude of pVW–MSCs was partially reduced by NaHS: the cells maintained the ability to grow in spheroid and sprouting from that, but endothelial markers (Factor VIII and CD31) were reduced. In conclusion, NaHS can be toxic for pVW–MSCs in high doses, while in low doses, it influences cellular physiology, by affecting the gene expression with a slowing down of the endothelial lineage.

## 1. Introduction

Although hydrogen sulfide (H_2_S) has long been known for its toxicity, now it is well documented as a endogenously produced gas transmitter, which would act as a cell modulator as nitric oxide (NO) and carbon monoxide (CO) [1,2,3,4].

In order to cast light on the physiological H_2_S properties, many different inhibitors of H_2_S synthesis or exogenous donors have been used. Among these donors, NaHS and Na_2_S were often used in in vitro studies, [5]. Nevertheless, opposite results were obtained by using these donors, depending on the cellular type [3,6]. Furthermore, in aqueous solution, three distinct chemical species coexist, namely H_2_S, HS^-^ and S^2-^, whose chemical interconversion is pH dependent. At physiological pHs, the S^2-^ concentration is insignificant, but H_2_S and HS^-^ coexist in a proportion which varies according to the different ambient conditions. Since the two species can activate different signaling pathways [7], the same nominal H_2_S concentration can lead to different effects in different cells. Accordingly, cytoprotective effects were demonstrated in human, mouse and rat cardiomyocytes [8], and on the contrary, cell death was detected in rat intestinal epithelial cells [9] and in human lung fibroblasts [10]. The toxicity of H_2_S donors seems to be due to a DNA-damaging mutagen property [11], as demonstrated in glioblastoma (GBM) cells, where NaHS interferes with cell cycle phases. The genotoxic effects could involve autoxidation mechanisms that generate reactive oxygen species (ROS) radicals [11,12] or mechanisms that impair DNA repairing via post-translational sulfhydration [13].

Furthermore, recent studies suggested that H_2_S could be involved in stem cell physiology [14,15,16,17]. In detail, mesenchymal stem cells (MSCs) were shown to produce H_2_S, whose endogenous synthesis plays an important role in the maintenance of cellular functions and homeostasis [17]. In pathological models, the treatment with H₂S donors could increase the proliferation and survival of MSCs attenuating hypoxia-induced apoptosis by enhancing the antiapoptotic gene expression [18,19]. Moreover, it has also been reported that H₂S improved the survival of transplanted MSCs into infarcted myocardium in a rat model [20] and exerted an antifibrosis role in cardiovascular disease [21].

The blood vessel is a source of many multipotent progenitor cells. Among these, vascular wall–mesenchymal stem cells (VW–MSCs) have been isolated from the tunica media and adventitia of different large vessels, including aorta, in many different species [22].

The VW–MSCs not only showed classical mesenchymal trilineage potential, but also differentiated toward endothelial phenotype and sustained the formation of capillary networks [23,24,25,26]. To date, the role of VW–MSCs in vascular regeneration has been intensely studied [27] and VW–MSCs are now emerging as a powerful tool for studying angiogenesis in physiological and pathological contexts [28].

Vascular wall–mesenchymal stem cells (VW–MSCs) have been isolated and characterized from the thoracic aorta of pig [29], an excellent animal model for translational purposes for its high anatomical, physiological and metabolic similitudes with humans. pVW–MSCs and their secreted factors possess a proangiogenic attitude [26,29], in particular the secretome of pVW–MSCs, is enriched with bioactive factors, such as growth factors, cytokines and chemokines. These many factors enhance in vitro angiogenesis [30], and promote neuronal remodeling, thus confirming the paramount role of this cell type in modulating immune-mediated response [31].

Taking into account all these data and considering the lacking information about the H_2_S role in vascular stem cells, the present research aimed at investigating the effect of a commonly used H_2_S donor, NaHS, on porcine vascular wall–mesenchymal stem cells in the intriguing perspective of a potential use of this putative cell modulator in the field of regenerative medicine.

## 2. Results

### 2.1. NaHS Effects on pVW–MSC Viability

**Figure 1:**
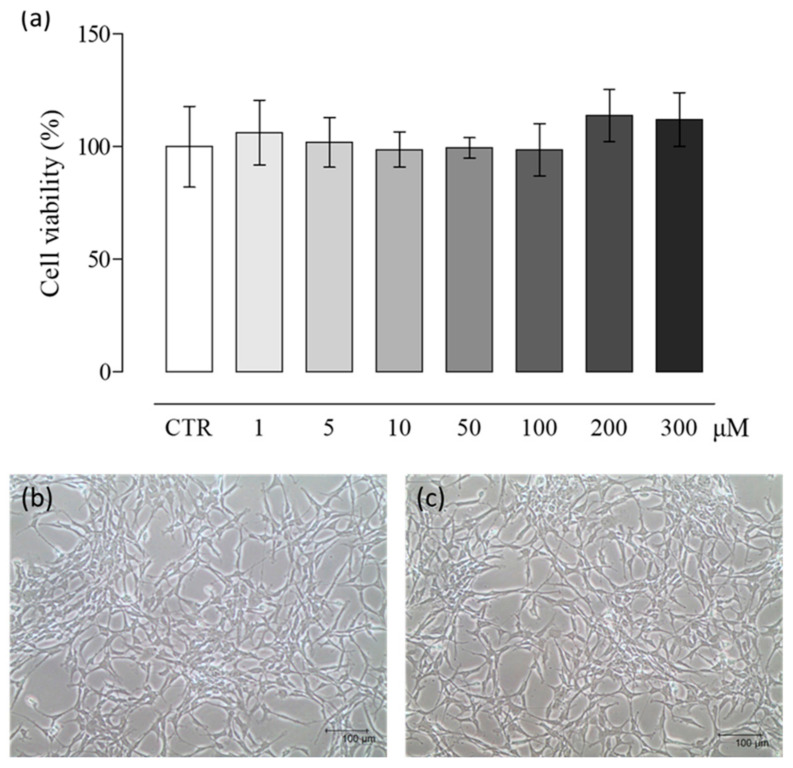
NaHS effect on the vascular wall–mesenchymal stem cell (VW–MSC) viability. Cells were treated with different doses of NaHS for 24 h and cellular viability was measured by MTT assay (control (CTR) = DMSO 0.01% *v*/*v*). (**a**) Data represent the mean ± S.D. of three independent experiments. (**b**) Representative images of cell morphology in the control cells and (**c**) after 24 h of NaHS 300 μM treatment. Scale bar = 100 µm

The effects of various NaHS concentrations on pVW–MSC viability were evaluated. MTT results demonstrated no toxicity in the NaHS concentration range studied (Figure 1a). Accordingly, no change in morphology was pointed out: the cells showed a small cell body with the very long prolongations typical of vascular stem cells, both in control (Figure 1b) and treated samples (Figure 1c).

### 2.2. NaHS Effects on pVW–MSC Cell Cycle and ROS Production

**Figure 2:**
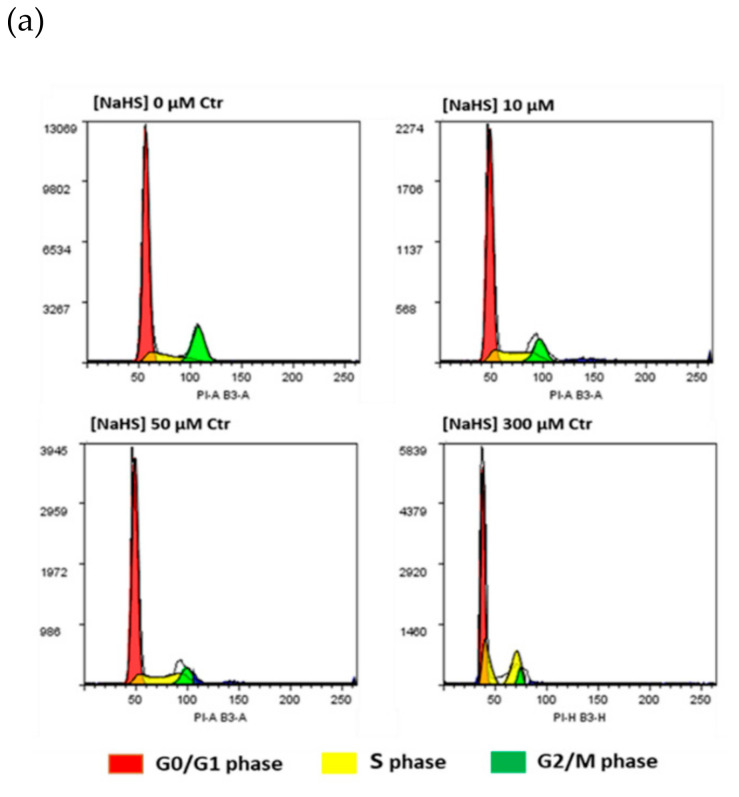

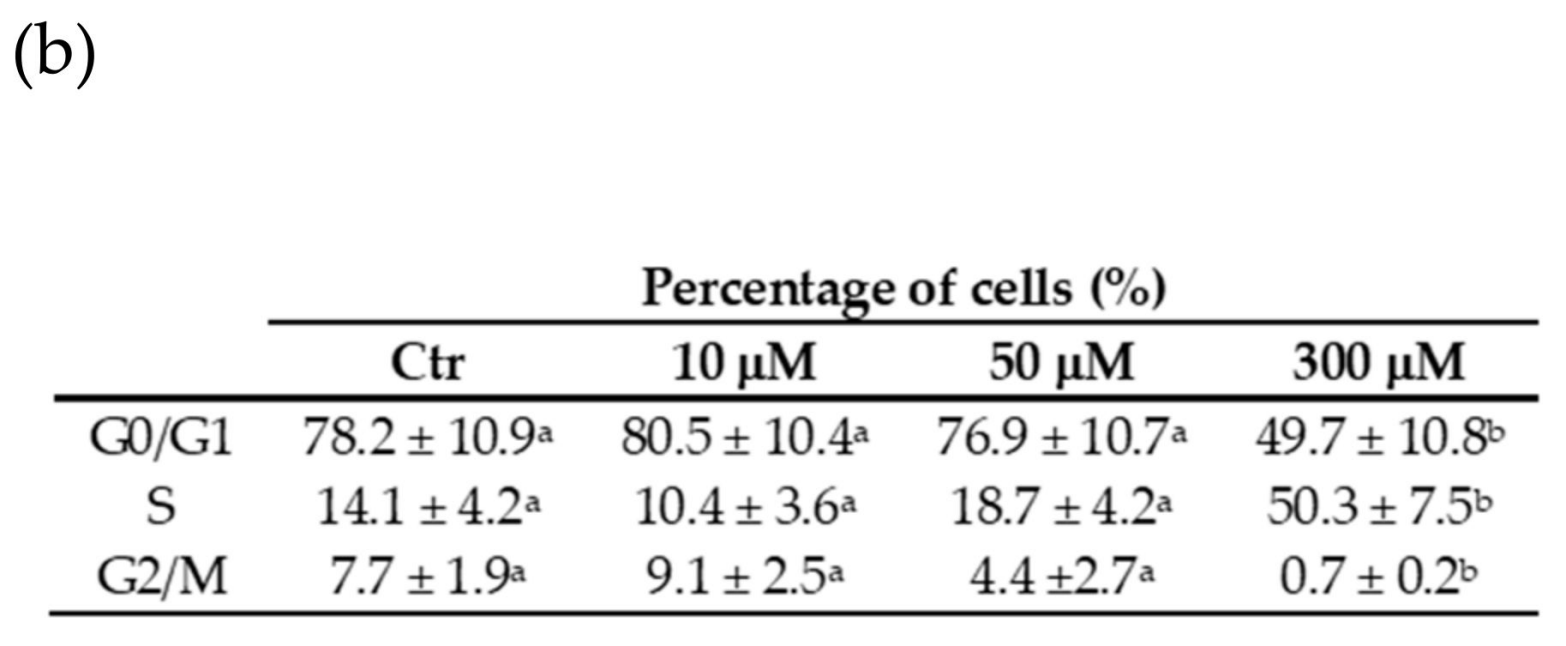
Effect of NaHS increasing doses (0, 10, 50, 300 μM) on the pVW–MSC cell cycle after 24 h of treatment (CTR = DMSO 0.01% *v*/*v*). (**a**) Representative histograms. (**b**) Percentage of cells (mean ± S.D.) of each phase at the different doses. Results are representative of three independent experiments; different superscript letters indicate significant differences among groups (*p* < 0.05, one-way ANOVA, post hoc Tukey comparison test).

The potential NaHS effects on pVW–MSC cell cycle were evaluated by the cytofluorimetric investigation of DNA content. The results showed that NaHS treatment at the highest dose (300 µM) affected the cells’ distribution in cell cycle phases: in detail, the G0/G1 ratio extensively decreased, most of cells were arrested in S phase, while the G2/M phase was substantially absent. Conversely, at the low NaHS doses, no effects on cell cycle were shown (Figure 2).

**Figure 3:**
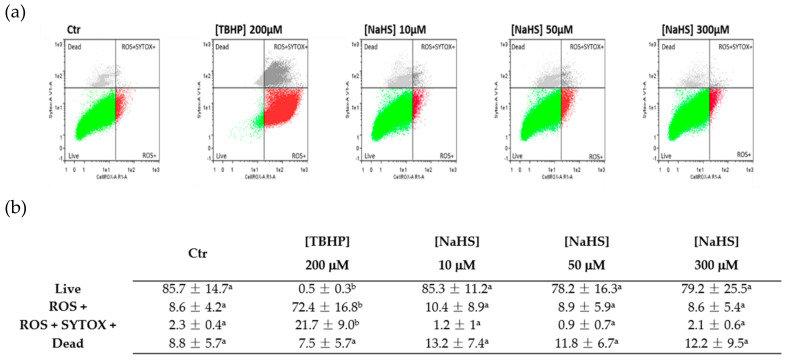
Representative graphs of reactive oxygen species (ROS) evaluation by flow cytometry in the pVW–MSCs treated with increasing doses of NaHS (0, 10, 50, 300 μM) after 24 h (CTR = DMSO 0.01% *v*/*v*) (**a**); green: live cells, red: ROS+ cells, light grey: dead cells and dark grey: ROS+ SYTOX+ cells. (**b**) Percentage of cells (means ± S.D) at each dose. Results are representative of three independent experiments; different superscript letters indicate significant differences among groups (*p* < 0.05, one-way ANOVA, post hoc Tukey comparison test).

To investigate whether the effect of NaHS on the cell cycle may involve ROS production, flow cytometric analysis using CellROX^®^ Deep Red Flow Cytometry Assay Kit was performed. Cell ROS analysis showed that NaHS did not induce ROS production: a basal ROS level occurred both in control cells and in all the NaHS-treated samples (Figure 3a, b). Tert-butyl hydroperoxide (TBHP), a strong ROS inducer, was used as a positive control.

### 2.3. NaHS Effects on pVW–MSC Mesenchymal Gene Expression and Angiogenic Property

**Figure 4:**
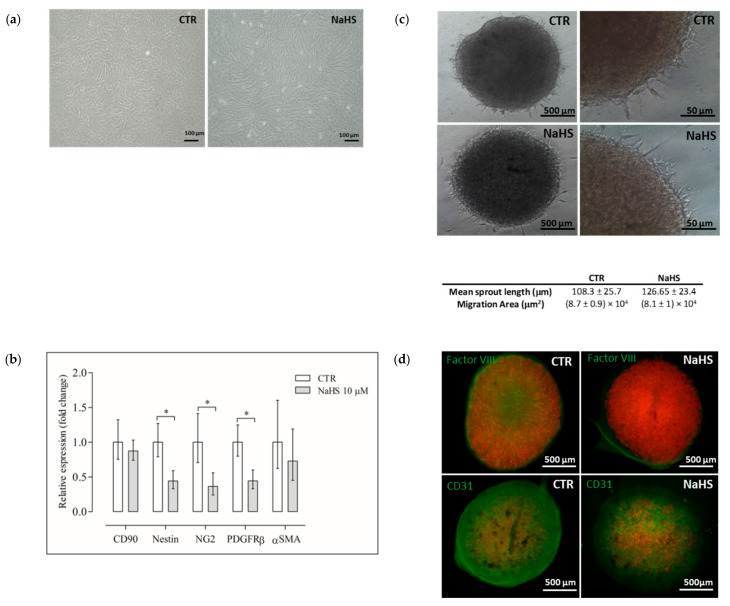
Effect of NaHS on the pVW–MSC gene expression profile and angiogenic attitude; (**a**) the representative images of pVW–MSCs morphology in control (CTR = DMSO 0.01% *v*/*v*) and NaHS-treated cells (10 μM) after 21 days of culture; scale bar = 100 μm; (**b**) the gene expression profile of some typical pVW–MSC markers CD90, Nestin, NG2, PDGFRβ, αSMA, in pVW–MSCs cultured 21 days with NaHS (10 μM) and their control (CTR). Data represent the mean ± the range of relative expression of three independent experiments. Data were analyzed using the Student’s *t*-test, significant differences are indicated as (*p* < 0.05). The relative expression (fold change) was calculated using the 2^−ΔΔCt^ method in relation to the control cells, (**c**) the representative images of pVW–MSC spheroids obtained through a 3D in vitro angiogenesis spheroid assay after 21 days of culture in control (CTR) and NaHS cell exposure (10 μM); the quantification of morphometric parameters: (the mean sprout length and the migration area) were reported, data represent the mean ± standard deviation of three independent experiments. The left images scale bar = 500 μm, right images scale bar = 50 μm; (**d**) the immunofluorescence analysis of pVW–MSC’s spheroids with endothelial markers Factor VIII and CD31(both green) in the control (CTR) and NaHS treatments (10 μM), and the nuclei were stained with propidium iodide (PI) (red) scale bar = 500 μm.

In order to ascertain whether the presence of NaHS in the culture medium could influence the characteristic genic profile of the pVW–MSCs, we cultured cells for 21 days with NaHS (10 µM), we decided to proceed with this safe dose to avoid any toxic effect. No differences in pVW–MSC morphology were shown after 21 days of culture with NaHS. All cells, in a confluent status, maintained their vascular-mesenchymal morphology (Figure 4a) [26]. Nevertheless, the expression of some characteristic VW–MSC markers (Nestin, NG2, PDGFR-β) were significantly decreased by the NaHS treatment (Figure 4b).

One of the most relevant features of VW–MSCs is their pro-angiogenic attitude. Accordingly, in this work, a 3D in vitro angiogenic spheroid assay was used to check NaHS effects on pVW–MSC angiogenic attitude. The sprouting of a typical cell appeared after 24 h in both the control and NaHS-treated spheroids (Figure 4c), but the immunofluorescence of Factor VIII and CD31 indicated that 10 µM NaHS reduced the endothelial markers expression (Figure 4d).

## 3. Discussion

Accumulating evidences show that H_2_S regulates many physiological and pathological processes, acting as a gas transmitter as NO and CO [1,2,3,4].

Recent interest has been addressed to the role of H_2_S in the field of mesenchymal stem cells (MSCs), so the use of H_2_S donors has been proposed to treat various pathological situations such as osteoporosis, immune disorders, inflammation and cardiovascular disease [17,18,19,20,21]. However, some H_2_S donors’ adverse effects were evidenced in different cell types [10,11,12], therefore, for the safe therapeutic use of these molecules, it is necessary to consider first that very different effects could depend on the context. In the present research, we investigated the effects of a well known H_2_S donor, NaHS, on vascular stem cells isolated from the porcine thoracic aorta (pVW–MSCs). Vascular Stem Cells (VSCs), are a promising class of stem cells in the field of regenerative medicine for their strong angiogenic capacities [22]. Lacking information regarding the role of this donor H_2_S in this particular class of vascular stem cells, the effect of NaHS on pVW–MSC viability and cell cycle was evaluated in a wide concentration range spanning from 1 to 300 µM. Even if NaHS did not apparently alter cell viability, we detected a deleterious effect on cell cycle at the highest dose (300 µM): the G0/G1 ratio extensively decreased, most of cells were arrested in S phase, while the G2/M phase was substantially absent. The NaHS ability to interfere with cell cycle was already demonstrated in other cell types, such as intestinal epithelial cells [32] and in human lung fibroblasts [10]. Oxidative stress was suggested as a potential mechanism of H_2_S-induced damage; however the ROS level in NaHS-treated pVW–MSCs, investigated in the present work, demonstrated that NaHS did not induce any increase in free radicals in pVW–MSCs, differently from reports in other cell types such HT-29-CI [11]. On the other hand, we previously demonstrated that pVW–MSCs are particularly resistant to oxidative stress since lipopolysaccharide (LPS), a common ROS inducer in many cellular types, did not cause oxidative stress in pVW–MSCs [33]. Therefore, in the present research, we hypothesized that NaHS is able to interfere with the cell cycle only at high doses, comparable with Na_2_S DE50% in producing genotoxic effects in CHO [32]. In our model, the damage is not mediated by ROS production, so further investigation will be necessary to clarify other possible DNA damage mechanisms.

We decided to proceed with a safe dose (10 µM) to verify if NaHS could influence the gene expression profile of pVW–MSCs, during a long-lasting culture time (21 days). Our results demonstrated that NaHS determined a significative reduction of Nestin, NG2 and PDGF-receptor β among the investigated staminal markers of pVW–MSCs. All these three markers are associated with pVW–MSC’s angiogenic capacity: PDGFR-β regulates the recruitment and proliferation of vascular cells by inducing the transcription and secretion of VEGF [34]; NG2 is a transmembrane proteoglycan protein, which was shown to increase in vivo angiogenesis [35]. Nestin, originally found in neuroepithelial stem cells, has been recently associated with a subset of perivascular MSCs [36], with strong angiogenic attitude.

Therefore, the impact of PDGFR-β, NG2 and Nestin reduction on the angiogenesis property of pVW–MSCs was investigated by a very powerful tool: the 3D in vitro angiogenic spheroid assay [37], demonstrated that, even if cellular sprouting was evident in both control and NaHS treated spheroid, the expression of endothelial markers (Factor VIII and CD31) was strongly reduced by the H_2_S donor.

Our results are partially in contrast with the H_2_S angiogenic property demonstrated for other cell types: H_2_S has been reported to stimulate endothelial cell proliferation, migration, and angiogenesis [38,39,40]. However, in our model, H_2_S did not completely disrupt the angiogenesis property of pVW–MSCs, in fact, the cells maintained the ability to grow in cellular aggregates and then, these spheroids could sprout if cultured on an extracellular matrix. Nevertheless, NaHS interfered with pVW–MSC endothelial differentiation. The H_2_S ability to influence cell differentiation, described in other stem cell populations [14,41], was hypothetically ascribed to the capacity to alter cellular signaling pathways by the sulfhydration of target proteins [42,43]. pVW–MSCs show a very high differentiation potential, as they are able, under the specific stimulation, to differentiate towards the three mesodermal lineages (osteo-, chondro- and adipo-) as well as into endothelial and smooth muscle cells [26,29]. This allows us to speculate that other types of differentiation could be favored by the NaHS treatment in pVW–MSCs, and in the intriguing perspective of a potential use of H_2_S donor in regenerative medicine, this deserves to be investigated in further investigation.

In conclusion, as far as we are aware, this is the first paper that explores the effects of the H_2_S donor, NaHS, on an emerging class of vascular stem cells: the vascular wall mesenchymal stem cells. To sum up, NaHS can be toxic in high doses while in low doses it influences pVW–MSC cellular physiology, by affecting the gene expression with a slowing down of the endothelial lineage.

## 4. Materials and Methods

### 4.1. Chemicals and Reagents

Dulbecco phosphate buffered saline (DPBS), phosphate buffered saline (PBS), human endothelial serum free medium (hESFM), a CellROX™ Deep Red Flow Cytometry Assay Kit, RNaseA/T1, TRIzol reagent and the GeltrexTM LDEV-Free Reduced Growth Factor Basement Membrane Matrix were purchased from Thermo Fisher Scientific (Thermo Fisher Scientific, Waltham, MA, USA). Propidium iodide (PI) was purchased from Miltenyi Biotec (Miltenyi Biotec, Bergisch Gladbach, Germany). RNA isolation was performed with the NucleoSpin RNA II kit (Macherey-Nagel GmbH & Co. KG, Düren, Germany). iScript cDNA synthesis kit, iTaq Universal SYBR Green Supermix and iTaq Universal Probe Supermix were used for the cDNA synthesis and qPCR analysis, all these products are by Bio-Rad (Bio-Rad Laboratories Inc., Hercules, CA, USA). The 3-(4,5-dimethylthiazol-2-yl)-2,5-diphenyltetrazolium bromide (MTT) and dimethyl sulfoxide (DMSO) were purchased from Sigma-Aldrich (Sigma-Aldrich, St. Louis, MO, USA). Pericyte growth medium (PGM) was purchased from PromoCell (PromoCell GmbH, Heidelberg, Germany). Sodium hydrogen sulfide (NaHS) was purchased from Cayman (Cayman Chemical, Ann Arbor, MI, USA). All plastic supports for cell culture were purchased from Corning-Becton, Dickinson (Corning-Becton, Dickinson and Company Becton Drive, Franklin Lakes, NJ, USA).

### 4.2. Cell Cultures

Primary cell cultures of porcine vascular wall–mesenchymal stem cells (pVW–MSCs) were isolated and maintained as previously described [26]. pVW–MSCs at the third passage were used in all experiments. The cells were seeded and routinely cultured in primary culture flasks (1.5 × 10^5^ cells/T75-flask) in PGM, with 1× antibiotic–antimycotic solution in a 5% CO_2_ atmosphere at 38.5 °C. Sodium hydrogen sulfide (NaHS), supplied as a crystalline solid, was dissolved in dimethyl sulfoxide (DMSO). Appropriate stock solutions were prepared solubilizing NaHS in DMSO at different concentrations. Each stock solution was then diluted 100× in culture media for cell exposure, obtaining the final NaHS doses of 0, 1, 5, 10, 50, 100, 200, 300 μM and a fixed DMSO concentration (0.01% *v*/*v*). The same amount of DMSO (0.01% *v*/*v*) was used as control vehicle.

### 4.3. Cell Viability

pVW–MSCs were seeded in a 96-well plate (3 × 10^3^/well) and the day after, were treated with NaHS at increasing doses (0, 1, 5, 10, 50, 100, 200, 300 μM) for 24 h. The MTT assay was performed at the end of the treatment time following the manufacturer’s instructions. Briefly, the MTT substrate was added to the culture medium and incubated for 4 h, then the MTT solubilization solution was added to the cells to dissolve the formazan crystals. Formazan Abs were measured at 570 nm, using Infinite^®^ F50/Robotic absorbance microplate readers from TECAN Life Sciences (Männedorf, Switzerland). Cell morphology was routinely checked using an inverted Eclipse Microscope (TS100) equipped with a digital C-Mount Nikon phothocamer (TP3100)

### 4.4. Cell Cycle Analysis

pVW–MSCs were seeded in T25-flask (1 × 10^6^ cells) and the day after, treated with increasing doses of NaHS (0, 10, 50, 300 µM) and incubated for 24 h. The cells were harvested and counted. Aliquots of 8 × 10^5^ cells were washed twice in 5 mL of PBS and fixed overnight in 70% ice-cold ethanol (800 µL), added drop by drop with continuous vortexing. Then, the cells were washed with 10 mL of PBS and the cellular pellet was incubated with 800 µL of staining solution containing 50 µg/mL of PI and 100 µg/mL RNaseA/T1 in PBS for 30 min in the dark at room temperature. Cell distribution in cell cycle phases was analyzed by MACSQuant^®^ Analyzer10 and Flowlogic software (Miltenyi Biotec, Bergisch Gladbach, Germany). Cellular events were discriminated from debris using forward scatter area (FSC-A) and side scatter area (SSC-A). Doublets were excluded for analysis by FSC-area and height (FSC-A/FSC-W). The Dean–Jett–Fox univariate model was used to determinate the percentage of the cell population in the different phases of the cell cycle [44].

### 4.5. Reactive Oxygen Species Evaluation

pVW–MSCs were seeded in a T-25 flask (1 × 10^6^) and the day after, treated with NaHS (0, 10, 50 and 300 μM) and incubated for 24 h. TBHP (200 μM), was used as a ROS inducer (positive control). At the end of the treatment, the cells were harvested, counted and the CellROX^®^ Deep Red Flow Cytometry Assay Kit was performed following the manufacturer’s instructions. Briefly, aliquots of 2 × 10^5^ cells/mL were stained with the kit-provided fluorescent probes. The samples were analyzed on MACSQuant Analyzer 10 (Miltenyi Biotec, Bergisch Gladbach, Germany) equipped with 638 nm lasers and 405 nm for the excitation of CellROX^®^ Deep Red and SYTOX^®^ Blue fluorescence, respectively. The data analysis was performed using Flowlogic software (Miltenyi Biotec, Bergisch Gladbach, Germany). Cellular events were discriminated from debris using forward (FSC-A) and side scatter (SSC-A) areas. Doublets were excluded from analysis by the FSC-area and height. CellROX^®^ Deep Red signal (excitation/emission: 644/655) was collected in the R1 channel (Filter 655–730 nm) and the SYTOX^®^ Blue signal (excitation/emission: 444/480) in the V1 channel (450/50 nm). Gates applied for population discrimination were manually set based on control samples.

### 4.6. Gene Expression Analysis after Long Term Culture with NaHS

pVW–MSCs were seeded in a 24-well plate (3 × 10^4^ cells/well), 24 h after the cell seeding, and NaHS (0, 10 μM) was added to the culture. The pVW–MSCs were cultured for 21 days in the presence or absence of NaHS, at each change of the medium the NaHS treatment was repeated. After 21 days, the pVW–MSCs were lysed then the RNA extraction and cDNA synthesis were carried out as previously described [45]. The gene expression analysis of VW–MSC markers (thy-1 cell surface antigen (CD90), Nestin, neuron-glial antigen 2 (NG2), platelet-derived growth factor receptor β (PDGFRβ) and α-smooth muscle actin (αSMA)) was performed by quantitative real time PCR (qPCR) as previously reported by us [26]. The gene expression analysis was conducted as previously reported by using two different reference genes (β-Actin (β-Act) and glyceraldehyde 3-phosphate dehydrogenase (GAPDH)) [45]. Primer sequences, expected PCR product lengths and accession numbers are reported in Table 1. The expression level of the interest gene (GI) was determined using the 2^−∆∆Ct^ method [46] in which ∆Ct = (Ct interest gene − Ct mean ref. genes) and ∆∆Ct = ∆Ct NaHS group − ∆Ct Control group.

### 4.7. In Vitro Angiogenesis Spheroid Assay

pVW–MSCs were seeded in the T-75 flask (1.5 × 10^6^) in PGM, 24 h after the cell seeding, and NaHS (0, 10 μM) was added to the culture. pVW–MSCs were cultured for 21 days in the presence or absence of NaHS, and at each change of the medium, the NaHS treatment was repeated. After 21 days, the cells were detached, counted, and the spheroids were produced by hanging drop technique [37]. Briefly, the pVW–MSCs were resuspended at a 6 × 10^4^/10 µL concentration. The petri tissue culture dish of 60 mm was used to perform the experiment. DPBS (5 mL) was placed at the bottom of the dish to create a hydration chamber. Drops of 1.2 × 10^5^ cells in 20 µL of PGM medium were seeded on the cover of the dish. Then, the cover was overturned and incubated at 5% CO_2_, 38.5 °C. After 24 h, the spheroids were transferred in an eight-well chamber slide, pretreated with 100 µL/well of GeltrexTM LDEV-Free Reduced Growth Factor Basement Membrane Matrix and cultured in hESFM (human endothelial serum-free medium) supplemented with 5% FBS, 1× antibiotic–antimycotic, and 50 ng/mL of hVEGF medium for 3 days in the presence or absence of NaHS (10 µM). Spheroids were monitored for sprouting formation. At the end of treatment, they were washed with DPBS and fixed in 4% paraformaldehyde for 1 h at room temperature, then permeabilized with 0.3% Triton-X 100 and washed three times in PBS 1× for 5 min. For non-specific sites, blocking slides were treated with 10% normal goat serum in PBS 1× for 1 h at RT then incubated overnight in a humidified chamber with the appropriate primary antibody dilution (Table 2). In negative controls, the primary antibody was omitted. Then, the samples were washed three times in PBS 1×, incubated with the appropriate dilution of a secondary antibody (Table 2) for 1 h at RT in a humidified chamber. After two washes for 5 min in PBS, 1× coverslips were mounted on slides with Fluoreshield with PI (Sigma-Aldrich, St. Louis, MO, USA). Photomicrographs were acquired using an Eclipse E600 epifluorescence microscope equipped with a Nikon digital camera and ACT-2 U software for image capturing (Nikon, Tokyo, Japan).

### 4.8. Statistical Analysis

Each treatment was replicated three or eight times (viability test) in three independent experiments. Data were analyzed by the Student’s *t*-test, or by one-way analysis of variance (ANOVA) followed by the post hoc Tukey comparison test. Differences of at least *p* < 0.05 were considered significant. Statistical analysis was carried out using Graph Pad Prism 7 software.

## Figures and Tables

**Table 1:** Primer Sequences Used for the qPCR Analysis.

Genes	Sequence (5′–3′)	PCR Product (bp)	Accession Number	Reference
CD90	For: GACTGCCGCCATGAGAATAC	180	NM_001146129.1	Zaniboni et al., 2015
	Rev: GGTAGTGAAGCCTGATAAGTAGAG			
Nestin	For: CAGTGGTTCCAAGGCTTCTC	163	ENSSSCT00000027298	Zaniboni et al., 2015
	Rev: CATAGGTGTGTCAAGGGTATCG			
NG2	For: ACCACCTCCTCCTACAACTC	104	ENSSSCT00000002098	Zaniboni et al., 2015
	Rev: GTCACTCAGCAGCATCTCTG			
PDGFRβ	For: GCAACGAGGTGGTCAACTTC	111	ENSSSCT00000015788	Zaniboni et al., 2015
	Rev: GCAGGATAGAACGGATGTGG			
αSMA	For: CACGGCATCATCACCAACTG	200	NM_001164650	Zaniboni et al., 2015
	Rev: ACCGCCTGAATAGCCACATAC			
GAPDH	For: ACATGGCCTCCAAGGAGTAAGA	106	NM_001206359	Tubon et al., 2019
	Rev: GATCGAGTTGGGGCTGTGACT			
	Probe: HEX-CCACCAACCCCAGCAAGAGCACGC-BHQ1			
β-Actin	For: CTCGATCATGAAGTGCGACGT	114	KU672525.1	Tubon et al., 2019
	Rev: GTGATCTCCTTCTGCATCCTGT			
	Probe: FAM-ATCAGGAAGGACCTCTACGCCAACACGG-BHQ1			

**Table 2:** Antibodies Used for the Immunofluorescence Analysis on the Spheroids.

Antibody	P. Number	Species	Supplier	Dilution
Anti CD31	MCA1746	Mouse	AbD Serotec	1:50
Anti Factor-VIII	RFF-8C/8	Mouse	BioRad	1:50
Anti-Mouse-FITC	F4143	Goat	Sigma-Aldrich	1:200

## Abbreviations

H_2_S	Hydrogen sulfide
VW–MSCs	Vascular wall–mesenchymal stem cells
CD90	THYmocyte differentiation antigen 1
NG2	Neural/glial antigen 2
PDGFR-β	Platelet-derived growth factor receptor beta
α-SMA	Alpha-smooth muscle actine
pVW–MSCs	Porcine vascular wall–mesenchymal stem cells
CD31	Platelet endothelial cell adhesion molecule
NO	Nitric oxide
CO	Carbon monoxide
ROS	Reactive oxygen species
VSCs	Vascular stem cells
